# Effectiveness and Feasibility of Pharmacist-Driven Penicillin Allergy De-Labeling Pilot Program without Skin Testing or Oral Challenges

**DOI:** 10.3390/pharmacy9030127

**Published:** 2021-07-20

**Authors:** You-Chan Song, Zachary J. Nelson, Michael A. Wankum, Krista D. Gens

**Affiliations:** Department of Pharmacy, Abbott Northwestern Hospital, Mail Route 11321, 800 East 28th Street, Minneapolis, MN 55407, USA; Zachary.Nelson2@allina.com (Z.J.N.); Michael.Wankum@allina.com (M.A.W.); Krista.Gens@allina.com (K.D.G.)

**Keywords:** penicillins, hypersensitivity, pharmacists, Antimicrobial Stewardship, pharmacy services, allergy

## Abstract

Documented penicillin allergies have been associated with an increased risk of adverse outcomes. The goal of this project was to assess the effectiveness and feasibility of a pharmacist-led penicillin allergy “de-labeling” process that does not involve labor-intensive skin testing or direct oral challenges. Adult patients with penicillin allergies were identified and interviewed by an infectious diseases pharmacy resident during a 3-month pilot period. Using an evidence-based standardized checklist, the pharmacist determined if an allergy qualified for de-labeling. In total, 66 patients were interviewed during the pilot period. The average time spent was 5.2 min per patient interviewed. Twelve patients (18%) met the criteria for de-labeling and consented to the removal of the allergy. Four patients (6%) met the criteria but declined removal of the allergy. In brief, 58.3% of patients (7/12) who were de-labeled and 50% of patients (2/4) who declined de-labeling but had their allergy updated to reflect intolerance were subsequently prescribed beta-lactam antibiotics and all (9/9, 100%) were able to tolerate these agents. A pharmacist-led penicillin allergy de-labeling process utilizing a standardized checklist is an effective and feasible method for removing penicillin allergies in patients without a true allergy.

## 1. Introduction

In the US, 10% of the population report allergies to penicillin (PCN) [1]. True hypersensitivity is uncommon and some studies suggest up to 95% of patients who report penicillin allergies are able to tolerate this class of medications [2]. Documented penicillin allergies can lead to increased health risks. Studies have shown that documented penicillin allergies are associated with an increased risk of Methicillin resistant *Staphylococcus aureus* (MRSA) and *Clostridioides difficile* (*C. difficile*) infections [3]. Another study demonstrated that a reported penicillin allergy increased the risks of surgical site infections as a result of patients not receiving first-line peri-operative antibiotics [4]. “De-labeling” of inappropriately documented allergies can decrease the use of unnecessary broad-spectrum antibiotics [5] and potentially prevent negative patient outcomes.

Documented penicillin allergies can also have a negative impact on infants born to mothers who received inadequate group B Streptococcal (GBS) prophylaxis. Clindamycin should be chosen as an alternative agent for intrapartum prophylaxis only when susceptibility has been confirmed. However, early-onset GBS infection in infants born from mothers receiving clindamycin has been reported due to increasing clindamycin resistance [6]. Use of inadequate GBS prophylaxis may also increase the observational period of neonates in the neonatal intensive care units to ensure that infants do not have invasive GBS disease [7,8].

Many studies have developed algorithms or questionnaires to aid in the process of de-labeling patients with PCN allergies [9,10]. Several algorithms used by different health systems across the US have been published and many recommend skin testing, direct oral challenges, and/or contain explicit recommendations for alternative cephalosporins [10]. Literature supports the use of penicillin antibiotics if the history suggests a non-immune mediated intolerance [3] or if the allergic reaction is considered low-risk [1]. Many penicillin allergies determined to be low-risk can be safely de-labeled without skin testing or oral challenges that often require a significant amount of time and resources [11]. In a recent study by Devchand, et al., 13% of patients screened for de-labeling were able to have their penicillin allergy label removed without any direct oral challenge or skin testing [5].

Early studies estimate about 10% of cephalosporins will exhibit cross-reactivity with penicillins [12]. However, these numbers are overestimated likely due to contamination of cephalosporin products with penicillin in earlier studies [13]. Further studies have revealed that the general rate of cross-reactivity with cephalosporins in patients with reported penicillin allergy is low (about 1%) and even in patients with a true penicillin allergy, the rate was significantly lower (about 2.5%) than the traditionally cited rate of 10% [14,15]. The key factor in determining cross-reactivity between penicillins and cephalosporins is the similarity of their R1 side-chains [16]. In general, many first and second generation cephalosporins possess similar R1 side chains with penicillins. Third and fourth generation cephalosporins can still be safely used in patients with IgE-mediated responses to penicillins due to dissimilarities in their R1 side-chains [15]. This evidence suggests that allergies and choices of agents should be guided on a case-by-case basis and cross-reactivity between penicillins and cephalosporins cannot be generalized as a class effect.

The primary objective was to assess the effectiveness (number of patients de-labeled) and feasibility (time spent) of a pharmacist-led process of penicillin allergy de-labeling without skin testing or direct oral challenges with penicillin agents.

## 2. Materials and Methods

The practice setting: This pilot was performed in select inpatient units at a tertiary community hospital. Two medical/surgical units and three labor/delivery and high-risk pregnancy units were chosen to test the primary objective.

**Figure 1:**
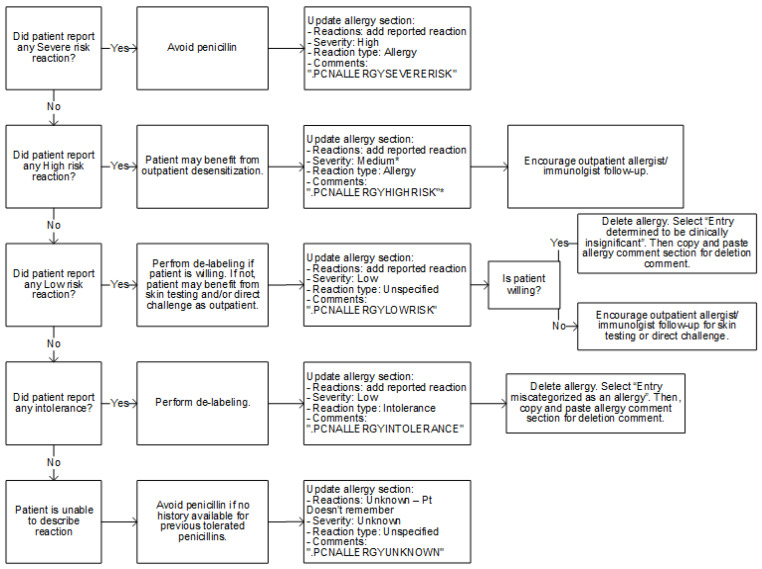
Approved algorithm for penicillin allergy de-labeling and outpatient referral.

Standardized checklist and de-labeling algorithm: The algorithm for penicillin allergy de-labeling is shown in Figure 1. This algorithm was adapted from an algorithm published in 2018 by Ramsey et al. in *The Journal of Allergy and Clinical Immunology: In Practice* [9]. A checklist (Supplementary Figure S1) for assessing penicillin allergy history was adapted from the Penicillin Allergy History Toolkit published in 2019 by Shenoy et al. [1] in *JAMA*.

**Figure 2:**
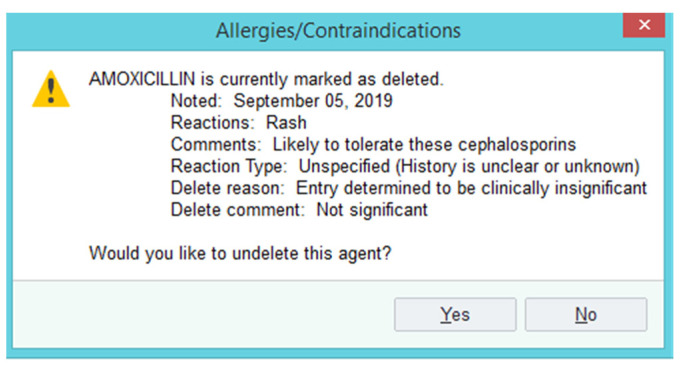
Alert to prevent re-addition of the allergy after deletion.

De-labeling process: A report within the electronic health record (EHR) was run once daily Monday through Friday to identify patients with documented penicillin allergies within the 3-month pilot period (23 October 2019–10 January 2020). Identified patients were interviewed by a post-graduate year two (PGY2) Infectious Diseases pharmacy resident and the allergy history was assessed utilizing the checklist in Figure S1. For “intolerance” or “low risk” reactions as identified in Figure S1, the penicillin allergy was modified to the appropriate reaction and reaction type (Figure 1). Documentation was added in the allergy section (as listed in Figure S2) and was also documented in a progress note (Figure S3). The penicillin allergy was then deleted from the EHR, preventing warnings from triggering upon order entry or verification of beta-lactam antibiotics. A deleted allergy is not viewable in the patient chart unless the user clicks “show deleted” within the allergy section. If a user tries to add the allergy again at a future date, an alert will display directing the user to the relevant documentation for review (Figure 2).

For all other reactions as identified in Figure S1, the penicillin allergy was modified to the appropriate reaction and reaction type (Figure 1). Documentation was added in the allergy section (as listed in Figure S2) and included in a progress note (Figure S3). In this case, the penicillin allergy was not deleted. All progress notes included the agents tolerated by the patient after the documented allergic reaction as well as a brief description and chart of beta-lactam cross-reactivity to help guide the selection of antibiotics in patients with true penicillin allergies (Figure S3). The interviewer provided the Penicillin Allergy Factsheet handout created by Centers for Disease Control and Prevention [12] to all patients to reinforce the importance of correctly identifying penicillin allergies and to encourage outpatient follow-up (not a formal referral) for skin testing and/or direct oral challenge if appropriate (as indicated in Figure 1).

A chart review was later performed in April 2020 to determine if any patients who underwent successful de-labeling during the pilot period were able to subsequently tolerate a beta-lactam antibiotic.

Inclusion criteria included: adult patients (18 years of age or older) with a documented penicillin allergy identified via an EHR report and admitted to one of two medical/surgical units or three labor/delivery and high-risk pregnancy units within the 3-month pilot period (23 October 2019–10 January 2020).

Documented allergies included: amoxicillin, ampicillin, ampicillin/sulbactam, amoxicillin/clavulanic acid, cloxacillin, dicloxacillin, floxacillin, nafcillin, oxacillin, penicillin, penicillin G, penicillin V, piperacillin, and piperacillin/tazobactam.

Exclusion criteria included: patients declining interviews and/or unable to be interviewed.

Primary outcome: effectiveness and feasibility of the pilot based on the percentage of de-labeled patients (effectiveness) and time spent by the pharmacist (feasibility). Effectiveness and feasibility were measured by the number of patients de-labeled and time spent during the patient interview, respectively. Univariable analysis was performed to display each outcome.

Secondary outcome: number of patients who were subsequently prescribed penicillins or cephalosporins (observational and not direct oral challenge).

## 3. Results

In total, 66 patients were interviewed during the pilot period. Twelve patients (18%) met the criteria for de-labeling and consented to the removal of the allergy from their chart. Four patients (6%) met the criteria for de-labeling but declined the removal of the allergy from their chart. (Table 1). The average time spent during patient interviews was 5.2 min per patient (Table 2). This time did not include the time utilized to perform a chart review prior to the interview and the documentation time after the interview was completed.

Upon chart review in December 2020, it was noted that seven patients (58.3%) were prescribed a beta-lactam after de-labeling and 100% (7/7) were able to tolerate the beta-lactam. Two patients who declined de-labeling but agreed to switch the allergy to an intolerance category were able to tolerate beta-lactam antibiotics as shown in Table 3.

## 4. Discussion

Literature documenting previous efforts in penicillin allergy de-labeling has been focused on skin testing and/or oral challenges to determine a patient’s true allergy status. One drawback of skin testing is the resource-intensive nature of the process [11]. The process usually takes about 2–3 h and the patient has to be monitored closely for potential allergic reactions. The result of this study provides a feasible and effective alternative. The development of the standardized checklist allows pharmacists to be highly efficient in conducting the interview. Average time for the interview process was about 5 min. This allows the interview process to potentially be incorporated into existing pharmacists’ medication reconciliation workflow, maximizing the number of patients that can undergo review for penicillin allergy de-labeling. Many health systems have incorporated pharmacist medication reconciliation processes to ensure the Center for Medicare and Medicaid Services (CMS) measures for admission medication reconciliation and transition of cares measures are met. Emphasizing the importance of collecting an accurate allergy history during this process and incorporating a standardized checklist may help institutions with limited resources establish a penicillin allergy de-labeling program within their practice.

There were many “low-hanging fruit” cases as seen in the case highlights (Table 4). Identifying a mislabeled allergy did not require an extensive interview, yet was an effective way to remove a documented penicillin allergy. Another case demonstrated how an incorrect allergy added to a young patient’s medical record may have led to the inappropriate prescribing of antibiotics in subsequent healthcare visits. This was prevented by de-labeling the mislabeled allergy using the standardized checklist. An additional benefit of this process was improved documentation of the allergy history that included previously tolerated beta-lactam agents. This information is especially useful when documented in the patient’s allergy profile. For patients who declined removal of the allergy despite meeting the “intolerance” category, the allergy was changed to an “intolerance” as appropriate. This also was an effective way to prevent warnings from triggering upon order entry and verification of beta-lactam agents. Lastly, the beta-lactam cross reactivity chart in the progress note serves as a guide to alternative antibiotic options with a low risk of cross-reactivity in patients with true penicillin allergies. One unique aspect of this pilot study was targeting patients in the maternity units; this population was chosen as many of these patients require aminopenicillin antibiotics for GBS prophylaxis. The initial goal was to perform a targeted interview prior to receiving prophylaxis antibiotics in patients who were GBS positive. However, there were logistical challenges that were identified during the pilot process. The intent of the pilot was to assess the feasibility of the process without interfering with routine patient care. Therefore, many patients were started on penicillin alternatives such as clindamycin prior to having an opportunity to interview with the pharmacist. Another challenge was that patients understandably did not want to get interrupted by staff who did not have a direct role in caring for them during and after labor. It was challenging to find an appropriate time to explain the process and perform the allergy assessment. Due to these barriers, providing an opportunity to have a penicillin allergy assessment completed during routine prenatal care visits may be a better approach in targeting this population.

There are several limitations to this study. First, this method cannot capture patients with a remote history of an allergic reaction. Studies have shown that 80% of the patients who have experienced an IgE-mediated reaction will lose sensitivity over the course of ten years [17]. It is likely that the majority of the patients reporting a remote childhood allergy are no longer allergic to penicillins. Second, due to the observational nature and limited time for follow-up, there was limited information available to determine if de-labeled patients were able to tolerate penicillin or a cephalosporin after de-labeling was performed. However, our study was still able to observe in subsequent encounters within our health system that all patients who received beta-lactam antibiotics after de-labeling were able to tolerate these agents with no complications (Table 3). Third, this is a single-center study and may not be applicable to other institutions. The baseline penicillin allergy rate was ~20% at our institution which is higher than the national reported average [18]. Fourth, this pilot was conducted by a single infectious diseases pharmacy resident and may have introduced a bias with a subjective interpretation of patients’ allergy history. This was mitigated by carefully reviewing and vetting the checklist with the hospital’s Antimicrobial Stewardship Committee consisting of ID physicians, ID pharmacists, and hospitalists. The checklist was designed to minimize variation among end users of the checklist. Furthermore, the checklist takes a very conservative approach in determining a high-risk reaction and does not define a timeframe for IgE-mediated allergic reactions. For example, a remote history (10+ years) of any IgE medicated reactions were considered ‘high risk’ per the checklist. However, existing literature suggests that more than 80% of these patients are not likely to have a penicillin allergy [1]. Lastly, there is still a possibility of a re-addition of the allergy after removal of the allergy. This problem was revealed when an inappropriate penicillin allergy label was added back to a patient’s chart at a subsequent clinic visit after the pharmacist performed de-labeling and de-labeled an amoxicillin allergy.

The 18% rate of de-labeling based on a targeted interview is similar to the rate of 13% reported by Devchand et al. given a relatively small number of data points in our study [5]. It is also worth noting that patient consent was required to remove a penicillin allergy once they met the criteria for de-labeling. The actual rate of de-labeling may be higher if protocol allowed for the removal an allergy in patients meeting criteria without pursuing consent. This proves that a pharmacist-led standardized checklist approach is an effective way to improve proper documentation of patients’ allergy histories. This can potentially decrease the risk of harmful outcomes such as MRSA infections, surgical site infections, and *C. difficile* infections in patients with documented penicillin allergies. In addition, several studies have also demonstrated that there is an economic burden in patients with documented allergies including increased antibiotic cost and increased lengths of stays in patients with documented penicillin allergies [19,20]. Further research is necessary to determine the potential patient and economic outcomes’ impact of this penicillin allergy de-labeling strategy.

## 5. Conclusions

A pharmacist-led penicillin allergy de-labeling process without skin-testing or oral challenges utilizing a standardized algorithm and checklist is an effective and feasible method in removing penicillin allergies in patients who do not have a true allergy to penicillins. Improved documentation of the allergy history and the information regarding the beta-lactam cross-reactivity serves as a useful tool in selecting safe alternative options in patients with true penicillin allergies. These strategies can help sites without the resources to conduct skin testing or direct oral challenges reduce the potential detrimental health and economic impacts that inappropriately documented penicillin allergies can have on patients. Further research is required to provide evidence to confirm the results of this pilot program.

## Figures and Tables

**Table 1:** Number of patients de-labeled.

	MB	Med/Surg	Total
De-labeled	4	8	12
Intolerance	1	3	4
True allergy	18	32	50
Total	23	43	66

‘MB’: Includes three labor/delivery and high-risk pregnancy units. ‘Med/surg’: Includes two medical/surgical units. ‘De-labeled’: patients met the criteria for de-labeling and the allergy was removed from the chart. ‘Intolerance’: patients met the criteria for de-labeling but declined the removal of the allergy from the chart. ‘True allergy’: patients who had a true allergy.

**Table 2:** Time spent during the patient interview.

	Time Spent (min)
Mean	5.2
Median	5
Range	12
Minimum	3
Maximum	15

**Table 3:** Patients tolerating a beta-lactam antibiotic after de-labeling (as of Dec. 2020).

Prescribed Antibiotics after De-Labeling/or Re-Labeling as Intolerance	Tolerated a Beta-Lactam Agent after De-Labeling	Agents Tolerated	
De-labeled 7/12 (58.3%)	7/7 (100%)	Amoxicillin/Clavulanate	1
		Piperacillin/Tazobactam	2
		Cephalexin	5
		Cefazolin	3
		Cefuroxime Axetil	1
		Ceftriaxone	3
		Cefepime	2
Intolerance 2/4 (50%)	2/2 (100%)	Ampicillin/Sulbactam	1
		Cefuroxime Axetil	2
		Cefdinir	1
Total 9/16 (56.3%)	9/9 (100%)		

**Table 4:** Case highlights.

**Patient 1: De-Labeling of an Amoxicillin Allergy** Patient reported a one-time GI reaction and some tingling after taking eight tablets of amoxicillin on an empty stomach prior to a dental procedure. Patient repeatedly reported during multiple inpatient/outpatient visits that he did not have an allergy but the allergy was never removed from the chart. Pharmacy resident removed the amoxicillin allergy after the interview. However, upon reviewing this patient’s case, penicillin allergy was added to patients’ chart during a subsequent provider visit. The patient tolerated piperacillin/tazobactam, cephalexin, and cefepime after de-labeling.
**Patient 2: Mislabeled Dicloxacillin Allergy** Patient had a documented dicloxacillin allergy. When asked about this allergy, patient denied the allergy and reported being allergic to doxycycline. De-labeling was performed and doxycycline was added to patient’s allergy list.

## Data Availability

Data is contained within the article or supplementary material.

## Supplementary Materials

The following are available online at https://www.mdpi.com/article/10.3390/pharmacy9030127/s1, Figure S1: Penicillin Allergy History Toolkit; Figure S2: Template to document in allergy section; Figure S3: Template progress note.
