# Supplementary material for: Effectiveness and Feasibility of Pharmacist-Driven Penicillin Allergy De-Labeling Pilot Program without Skin Testing or Oral Challenges
Source: Pharmacy (Basel). 2021 Jul 20;9(3):127. doi: 10.3390/pharmacy9030127 (PMC8293328; doi:10.3390/pharmacy9030127)
Supplement: Supplementary file 1 [file pharmacy-09-00127-s001.zip › pharmacy-1294894-supplementary.pdf]

Supplementary Material

Figure S1: Penicillin Allergy History Toolkit (Adapted from Shenoy et al. JAMA 321.2 (2019): 188-199)

Agent/Route:____________________ Date of Reaction ___________________

Reaction history (check all that apply):

Risk Level	Reaction	
Intolerance	☐ Isolated GI upset (diarrhea, nausea, vomiting, abdominal pain)<br>☐ Chills	☐ Headache<br>☐ Fatigue
Low risk	☐ Family history<br>☐ Itching WITHOUT rash (pruritus)	☐ Unknown, remote (>10 years ago) reaction<br>☐ Patient denies allergy but is on record
High risk (potential IgE reactions)	☐ Anaphylaxis<br>☐ Angioedema<br>☐ Bronchospasm (chest tightness)<br>☐ Cough<br>☐ Nasal symptoms<br>☐ Arrhythmia<br>☐ Throat tightness	☐ Hypotension<br>☐ Flushing/redness<br>☐ Shortness of breath<br>☐ Rash, type of rash (if known):_______<br>☐ Syncope/pass out<br>☐ Wheezing<br>☐ Dizzy/lightheadedness
Severe risk (potential severe none-immediate reactions)	☐ Steven-Johnson syndrome (rash with mucosal lesions)<br>☐ Serum sickness (rash with joint pain, fever, myalgia)<br>☐ Thrombocytopenia<br>☐ Fever (not from infection)<br>☐ Organ injury (liver, kidney)	☐ Erythema multiforme (rash with target lesions)<br>☐ Dystonia<br>☐ Anemia<br>☐ Acute generalized exanthematous (rash with pustules)<br>☐ Drug reaction eosinophilia and systemic symptoms (rash with eosinophilia and organ injury)
Other	☐ Other symptoms not listed above: ______________________	
Beta-lactam use	☐ Penicillin or beta-lactam use after documented reactions/allergy (patient reported vs chart review):<br>Patient reported:<br>Chart review:	

History Taken by: _____________________ Date: __________________

Figure S2: Template to document in allergy section

Smartphrase: ".PCNALLERGYUNKNOWN"

Infectious Diseases pharmacist completed allergy assessment. Unable to determine risk. Patient has tolerated [LIST PREVIOUS TOLERATED AGENTS] in the past. Penicillin Allergy Factsheet handout by CDC was given. See progress note from pharmacist on [DATE].

Smartphrase: ".PCNALLERGYINTOLERANCE"

Infectious Diseases pharmacist completed allergy assessment. Allergy determined to be intolerance. It is safe to use [AGENT] for this patient. Patient has tolerated [LIST PREVIOUS TOLERATED AGENTS] in the past. Will likely tolerate [CEPHALOSPORINS], as these do not share side chains and risk for cross-reactivity is low. Penicillin Allergy Factsheet handout by CDC was given. See progress note from pharmacist on [DATE].

Smartphrase: ".PCNALLERGYLOWRISK"

Infectious Diseases pharmacist completed allergy assessment. Allergy determined to be low-risk. Patient has tolerated [LIST PREVIOUS TOLERATED AGENTS] in the past. Will likely tolerate [CEPHALOSPORINS], as these do not share side chains and risk for cross-reactivity is low. Penicillin Allergy Factsheet handout by CDC was given. This patient may be a good candidate for direct oral challenge. See progress note from pharmacist on [DATE].

Smartphrase: ".PCNALLERGYHIGHRISK"

Infectious Diseases pharmacist completed allergy assessment. Allergy determined to be high-risk. Patient has tolerated [LIST PREVIOUS TOLERATED AGENTS] in the past. Will likely tolerate [CEPHALOSPORINS], as these do not share side chains and risk for cross-reactivity is low. Penicillin Allergy Factsheet handout by CDC was given. See progress note from pharmacist on [DATE].

Smartphrase: ".PCNALLERGYSEVERERISK"

Infectious Diseases pharmacist completed allergy assessment. Allergy determined to be high-risk. Do not recommend skin testing or challenge. Patient has tolerated [LIST PREVIOUS TOLERATED AGENTS] in the past. Will likely tolerate [CEPHALOSPORINS], as these do not share side chains and risk for cross-reactivity is low. Penicillin Allergy Factsheet handout by CDC was given. See progress note from pharmacist on [DATE].

Figure S3: Template progress note

Smartphrase: ".PCNALLERGYASSESSMENT"

Penicillin Allergy History Assessment

Assessment performed on [DATE] by [NAME, CREDENTIALS]

Patient name: ***
DOB: ***

Patient reported following reactions to [NAME OF PENICILLIN AGENT].

Intolerance: ***

Low risk: ***

Moderate-high risk: ***

High risk: ***

Other symptoms: ***

Penicillin Allergy Factsheet handout given: Y/N

Onset of symptoms after starting antibiotics was *** and patient received treatment with *** following the reaction. This reaction occurred *** (time frame) ago. PRIOR to this reaction, patient has tolerated ***. AFTER the reaction, patient has tolerated *** without complications.

Based on the history provided by the patient, this allergy has been deemed (low/intermediate/high) risk. Allergy list was updated to reflect this information.

Penicillin and cephalosporin allergies only show cross-reactivity if two agents have structural resemblance in side chains. Shown below are beta-lactam antibiotics with common side chains. Agents that do not share structural similarities in side chains do not have cross-reactivity. Refer to the table below and previously tolerated antibiotics in guiding antibiotic choice. This table was adopted from article by Zagursky et al. "Cross-reactivity in β-lactam allergy." Published in *The Journal of Allergy and Clinical Immunology: In Practice* 6.1 (2018): 72-81. to reflect products available in the US.

Date/time:
Name:
Phone: ***
Pager: ***

Penicillin and Cephalosporin Side Chains[3]

	Nafcillin	Oxacillin	Dicoxacillin	Penicillin G/V	Piperacillin	Ampicillin	Amoxicillin	Cefadroxil	Cephalexin	Cefazolin	Cefoxitin	Cefuroxime	Cefotetan	Cefprozil	Cefaclor	Ceftibuten	Cefdinir	Cefixime	Ceftriaxone	Cefditoren	Cefotaxime	Cefpodoxime	Ceftazidime	Cefepime	Ceftaroline	Ceftolozane
**Nafcillin**	■																									
**Oxacillin**		■	X																							
**Dicoxacillin**		X	■																							
**Penicillin G/V**				■	X	X	X	X	X					X	X											
**Piperacillin**				X	■	X (yellow)	X	X	X (yellow)					X	X (yellow)											
**Ampicillin**				X	X (yellow)	■	X	X	X (red)					X	X (red)											
**Amoxicillin**				X	X	X	■	X (red)	X					X (red)	X											
**Cefadroxil**				X	X	X	X (red)	■	X					X (red)	X											
**Cephalexin**				X	X (yellow)	X (red)	X	X	■					X	X (red)											
**Cefazolin**										■																
**Cefoxitin**											■	X (red)									X					
**Cefuroxime**											X (red)	■						X	X (yellow)	X (yellow)	X (yellow)	X (yellow)		X (yellow)	X	
**Cefotetan**													■													
**Cefprozil**				X	X	X	X (red)	X (red)	X					■	X											
**Cefaclor**				X	X (yellow)	X (red)	X	X	X (red)					X	■											
**Ceftibuten**																■	X (yellow)	X (yellow)	X (yellow)	X (yellow)	X (yellow)	X (yellow)	X (yellow)	X (yellow)	X	X
**Cefdinir**																X (yellow)	■	X (red)	X (yellow)	X (yellow)	X (yellow)	X (yellow)	X (yellow)	X (yellow)	X	X
**Cefixime**												X				X (yellow)	X (red)	■	X (yellow)	X (yellow)	X (yellow)	X (yellow)	X (yellow)	X (yellow)	X	X
**Ceftriaxone**												X (yellow)				X (yellow)	X (yellow)	X (yellow)	■	X (red)	X (red)	X (red)	X (yellow)	X (red)	X	X
**Cefditoren**												X (yellow)				X (yellow)	X (yellow)	X (yellow)	X (red)	■	X (red)	X (red)	X (yellow)	X (red)	X	X
**Cefotaxime**											X	X (yellow)				X (yellow)	X (yellow)	X (yellow)	X (red)	X (red)	■	X (red)	X (yellow)	X (red)	X	X
**Cefpodoxime**												X (yellow)				X (yellow)	X (yellow)	X (yellow)	X (red)	X (red)	X (red)	■	X (yellow)	X (red)	X	X
**Ceftazidime**																X (yellow)	X (yellow)	X (yellow)	X (yellow)	X (yellow)	X (yellow)	X (yellow)	■	X (yellow)	X	X (yellow)
**Cefepime**												X (yellow)				X (yellow)	X (yellow)	X (yellow)	X (red)	X (red)	X (red)	X (red)	X (yellow)	■	X	X
**Ceftaroline**												X				X	X	X	X	X	X	X	X	X	■	X (yellow)
**Ceftolozane**																X	X	X	X	X	X	X	X (yellow)	X	X (yellow)	■

X (red) = highest risk of cross-reactivity, fully identical side chains

X (yellow) = moderate risk of cross-reactivity, partially identical side chains

X = low risk of cross-reactivity, similarities in side chains

(blank) = no similarities in side chains, very low to no risk of cross-reactivity
